# Support Vector Machine-based Spontaneous Intracranial Hypotension Detection on Brain MRI

**DOI:** 10.1007/s00062-021-01099-x

**Published:** 2021-10-19

**Authors:** Philipp G. Arnold, Emre Kaya, Marco Reisert, Niklas Lützen, Philippe Dovi-Akué, Christian Fung, Jürgen Beck, Horst Urbach

**Affiliations:** 1 Department of Neuroradiology, Medical Center, University of Freiburg, Breisacher Str. 64, 79106 Freiburg, Germany; 2 Department of Medical Physics, Medical Center, University of Freiburg, Freiburg, Germany; 3 Department of Neurosurgery, Medical Center, University of Freiburg, Freiburg, Germany

**Keywords:** Bern score, Machine learning, Convolutional neural network, Cerebrospinal fluid leak, Magnetic resonance imaging

## Abstract

**Background and Purpose:**

To develop a fully automatic algorithm for the magnetic resonance imaging (MRI) identification of patients with spontaneous intracranial hypotension (SIH).

**Material and Methods:**

A support vector machine (SVM) was trained with structured reports of 140 patients with clinically suspected SIH. Venous sinuses and basal cisterns were segmented on contrast-enhanced T1-weighted MPRAGE (Magnetization Prepared-Rapid Gradient Echo) sequences using a convolutional neural network (CNN). For the segmented sinuses and cisterns, 56 radiomic features were extracted, which served as input data for the SVM. The algorithm was validated with an independent cohort of 34 patients with proven cerebrospinal fluid (CSF) leaks and 27 patients who had MPRAGE scans for unrelated reasons.

**Results:**

The venous sinuses and the suprasellar cistern had the best discriminative power to separate SIH and non-SIH patients. On a combined score with 2 points, mean SVM score was 1.41 (±0.60) for the SIH and 0.30 (±0.53) for the non-SIH patients (*p* < 0.001). Area under the curve (AUC) was 0.91.

**Conclusion:**

A fully automatic algorithm analyzing a single MRI sequence separates SIH and non-SIH patients with a high diagnostic accuracy. It may help to consider the need of invasive diagnostics and transfer to a SIH center.

**Supplementary Information:**

The online version of this article (10.1007/s00062-021-01099-x) contains supplementary material, which is available to authorized users.

## Introduction

Spontaneous intracranial hypotension (SIH) is an orthostatic headache syndrome that in almost all cases is caused by spinal cerebrospinal fluid (CSF) leaks [1]. Diagnostic criteria include a CSF pressure < 60 mm H_2_O and/or evidence of a CSF leak on imaging [2]; however, only approximately one third of SIH patients have a CSF opening pressure below 60 mm H_2_O, and the CSF opening pressure can, in fact, be normal or even elevated, particularly in patients with a long history of SIH [3, 4], or with large abdominal girth [5].

Among the numerous cranial MRI signs of SIH pachymeningeal enhancement (83%) and engorgement of the venous sinuses (93%) have the highest sensitivity; however, 20–30% of SIH patients are considered to have normal MRI scans [1, 6]. Engorgement of the venous sinuses does not only mean a volume plus but also a change of shape with, e.g. the inferior margin of the midportion of the dominant transverse sinus showing a distended convex appearance called the venous distension sign [7]. Dobrocky et al. 2019 proposed a scoring system which has been later termed the Bern score where pachymeningeal contrast enhancement, engorgement of venous sinuses and effacement of the suprasellar cistern of 4.0 mm or less were shown to be the most important discriminating features and weighted with 2 points each while subdural fluid collection, effacement of the prepontine cistern of 5 mm or less, and a mamillopontine distance of 6.5 mm or less were weighted with 1 point each resulting into a maximum score of 9 [8]. Patients with total scores of 2 points or fewer were classified as having a low, with 3–4 points as having an intermediate, and with 5 or more points as having a high probability for a spinal CSF leak; however, some of the features (especially the engorgement of the venous sinuses) are somewhat arbitrary in definition which can lead to a different assessment for different raters. We hypothesized that these and other shape changes of the venous sinuses are better detected by a machine learning algorithm. Therefore, in order to overcome such subjective visual assessments and to avoid time-consuming distance measurements we sought to develop a fully automatic classifier that discriminates SIH patients and healthy controls.

## Material and Methods

For training of a support vector machine (SVM) algorithm, we analyzed sagittal contrast-enhanced T1-weighted MPRAGE (Magnetization Prepared-Rapid Gradient Echo) sequences of 140 patients (149 MRI scans, female: 91, male: 58, mean age 44 ± 12 years) who were transferred to our clinic with suspected SIH between 2015 and 2020. They then received a standardized MRI protocol of the head and spine on a 1.5 (*n* = 120) or 3 (*n* = 29) Tesla scanner (Avanto/Trio/Prisma, Siemens Healthineers, Erlangen, Germany) and had structured reports with respect to the points of the SIH score proposed by Dobrocky et al. [8]. Of the 149 MRI scans 19 (12.8%) showed engorgement of the venous sinuses, 65 (43.6%) pachymeningeal enhancement, 39 (26.1%) effacement of the suprasellar cistern, 61 (40.9%) effacement of the prepontine cistern, 51 (34.2%) reduced mamillopontine distances and 20 (13.4%) subdural fluid collections.

The MRI protocol included axial T2-weighted, axial Diffusion Weighted Imaging (DWI), sagittal 3D-Fluid Attenuated Inversion Recovery (FLAIR) and sagittal contrast-enhanced MPRAGE sequences for the head and sagittal 3D T2-weighted SPACE (Sampling Perfection with Application optimized Contrasts using different flip angle Evolution) sequences for the spine.

### Segmentation of Volumes and Training of a Segmentation CNN

#### Venous Sinuses

The superior sagittal sinus was segmented from approximately 1–2 cm behind the coronal suture down to the confluens sinuum. The straight sinus was segmented in its entire length, and both transverse sinuses downwards to the transition to the sigmoid sinuses.

#### Basal Cisterns

**Fig. 1:**
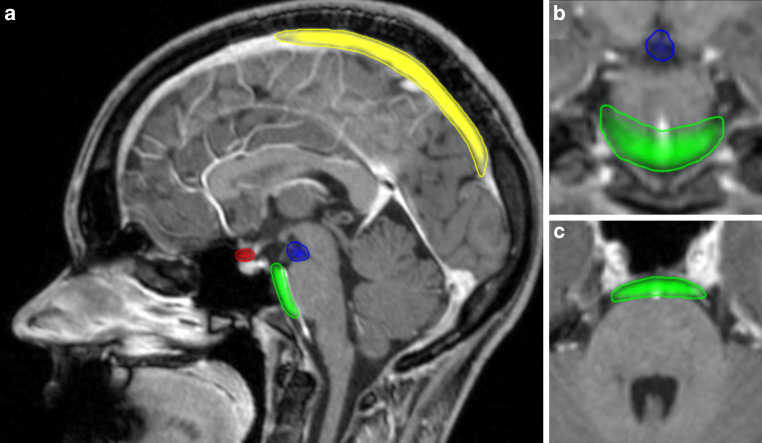
Midsagittal contrast-enhanced MPRAGE (Magnetization Prepared-Rapid Gradient Echo) slice (**a**) with automatically segmented suprasellar cistern (*red*), prepontine cistern (*green*), interpeduncular cistern (*blue*) and superior sagittal sinus (*yellow*) and coronal (**b**) and axial (**c**) projections

The prepontine cistern was segmented from the pontomesencephalic to the pontobulbar transition in craniocaudal direction and from the right to the left side so that the volume did not reach beyond the ventral pons surface. The suprasellar cistern was segmented from the pituitary gland upwards to the optic chiasm and from the ventral pituitary border to the pituitary stalk. The interpeduncular cisterns were segmented from the pontomesencephalic transition upwards to the mammillary bodies. Lateral borders were the cerebral peduncles (Fig. 1).

For training of the segmentation CNN, the abovementioned structures of 36 patients of the training cohort were manually segmented by an experienced neuroradiologist using the in-house developed postprocessing platform NORA (www.nora-imaging.com). The U‑net like neuronal network was implemented using the patchwork framework (https://bitbucket.org/reisert/patchwork/) and trained on contrast-enhanced T1-weighted MPRAGE scans. The CropGenerator was set to 3 dimensions (ndim = 3), the patch size to 32 × 32 × 32 voxels (“patch_size”: [32,32,32]) and the depth to 4 (depth = 4). The PatchworkModel itself was trained with 5 epochs (epochs = 5), 1000 iterations (num_its = 1000) and data augmentation was done with a random rotation angle of the patches dphi = 0.2 (“dphi”: 0.2). The output of the CNN is a voxel-wise probability value between 0 and 1 indicating whether the voxel belongs to the respective volume as displayed in Fig. 1.

The accuracy of the CNN segmentation was assessed with Dice coefficients. Thresholds showing the best overlap between manual and CNN segmentation were chosen for subsequent feature extraction (Suppl. Fig. 1a, b). As the Dice coefficient for the straight sinus was worse than that of the other sinuses, it was disregarded in the further analysis.

#### Feature Extraction

Eight radiomic 3D-shape features (major axis length, minor axis length, least axis length, elongation, flatness, surface area to volume ratio, sphericity, and volume) were extracted using the python library pyradiomics [9]. The dominant transverse sinus (ST-D) was determined by comparing the volumes of both transverse sinuses. In summary, we analyzed 32 (4 * 8) radiomic features for the sinuses, and 8 radiomic features for each cistern.

### SVM Classifier and SVM Training

For each of the automatically segmented volumes a SVM (python library scikit-learn [10]) was trained to discriminate absent (0) and present (1) SIH findings taking the structured reports as ground truth (GT). To determine the best combination of SVM parameters, i.e. C and gamma for radial basis function (RBF) kernels and C for linear kernels, in respect of area under the curve (AUC) a grid search approach was applied. Preprocessing was done by the StandardScaler to assure normal distribution of the input data. For the RBF kernel gamma was varied between 0.001 and 30 and C between 0.01 and 100, for the linear kernel C was varied between 0.001 and 750. The class_weight was set to “balanced”. To assess the sensitivity and specificity of the SVM results compared to the ground truth a leave-one-out cross-validation was performed. The assessments of the SVM results in terms of sensitivity, specificity, and accuracy for engorgement of the venous sinuses (Sinus), effacement of the prepontine cistern (PPC), effacement of the suprasellar cistern (SSC) and effacement of the interpeduncular cistern (IPC) are displayed in Table 1. As specificity was low, we disregarded the interpeduncular cistern for the validation. For each SVM a linear kernel with the respective C as shown in Table 1 value was used.

**Table 1:** Four-field tables comparing ground truth (GT) with SVM predictions (P)

	Sinus		PPC		SSC		IPC	
*n* = 149	P =0	P =1	P =0	P =1	P =0	P =1	P =0	P =1
GT = 0	*97* 65%	*33* 22%	*71* 48%	*17* 11%	*95* 64%	*15* 10%	*70* 47%	*28* 19%
GT = 1	*2* 1%	*17* 11%	*15* 10%	*46* 31%	*8* 5%	*31* 21%	*24* 16%	*27* 18%
Sensitivity	74.6%		80.7%		86.4%		71.4%	
Specificity	89.5%		75.4%		79.5%		52.9%	
Accuracy	76.5%		78.5%		84.6%		65.1%	
C	0.01		0.1		1		2.5	

Ground truth (GT) = 0 means that the sign was rated as absent in the structured reports, GT = 1 as present.

P = 0 (absent) and P = 1 (present) are the support vector machine (SVM) results.

*PPC* prepontine cistern, *SSC* suprasellar cistern, *IPC* interpeduncular cistern

## Results

For validation, we analyzed 34 patients (female: 25, male: 9, mean age 45 ± 10 years) with a CSF leak proven by conventional myelography, CT myelography or digital subtraction myelography (DSM) between 2018 and 2020 and no prior SIH-related treatment and 27 patients (female: 18, male: 9, mean age 46 ± 13 years) who underwent a contrast-enhanced T1-weighted MPRAGE sequence for unrelated reasons and had inconspicuous findings. Of the patients 27 (79.4%) of the SIH group had a CSF leak due to ventral dural tears, 5 (14.7%) patients due to leaking meningeal diverticulae, and 2 (5.9%) patients due to CSF venous fistulas. From the controls 27 patients were chosen so that they closely matched the age and the sex distribution of the CSF leak group.

**Fig. 2:**
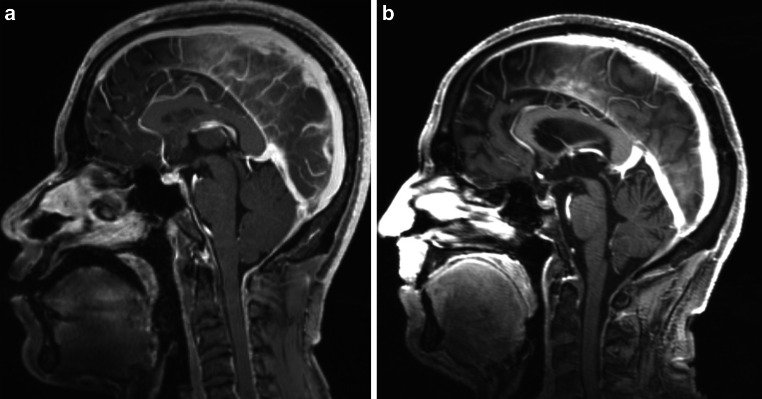
Sagittal contrast-enhanced MPRAGE (Magnetization Prepared-Rapid Gradient Echo) slices in a 34-year-old man with SIH due to a ventral dural tear at TH1/2 (**a**) and a 60-year-old man with an ocular myasthenia and a normal MRI (**b**). SVM-based SIH score is 2 in (**a**) and 0 in (**b**)

Among the segmented volumes, engorgement of the venous sinuses had the highest discriminative power to separate SIH patients and controls. The best discriminating radiomic features for the venous sinuses were the volume and the surface to volume ratio (Suppl. Fig. 2a and b). For the suprasellar cistern, the best discriminating radiomic features were least axis length and volume (Suppl. Fig. 3). The effacement of the prepontine cistern did not contribute to the discrimination between SIH-patients and normal controls, so it was disregarded for the calculation of the final SVM score. An example for midsagittal contrast-enhanced MPRAGE slices of a patient in the SIH group and the control group are shown in Fig. 2a, b.

**Fig. 3:**
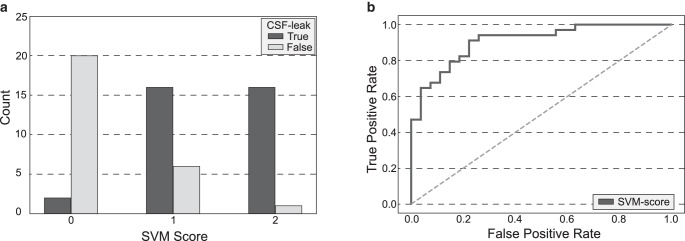
**a** SVM score for the validation cohort **b** ROC curve for SVM score consisting of the sum of the Sinus and SSC score. AUC is 0.91

The final SVM score consists of the sum of the Sinus and SSC score, leading to a maximum score of 2 (1 point for engorgement of the venous sinuses, 1 for effacement of the suprasellar cistern) and is shown in Fig. 3a for the validation cohort. The prepontine cistern was eliminated from the calculation of the SVM score due to its low specificity. The results for the SVM (i.e. Sinus + SSC), Sinus, SSC and PPC score are shown in Table 2.

**Table 2:** Results from the scoring of the validation cohort

Score	Sinus + SSC		Sinus		SSC		PPC	
	SIH	Controls	SIH	Controls	SIH	Controls	SIH	Controls
0 P	2	20	7	23	13	23	24	12
1 P	16	6	27	4	21	4	10	15
2 P	16	1	–	–	–	–	–	–
Mean	*1.41*	*0.30*	*0.79*	*0.15*	*0.62*	*0.15*	*0.29*	*0.56*

Two spontaneous intracranial hypotension (SIH) patients (5.9%) have 0 points (P), 16 patients (47.1%) 1 point and another 16 patients (47.1%) 2 points (mean: 1.41 ± 0.60), 20 non-SIH patients (74.1%) have 0 points, 6 patients (22.2%) 1 point and 1 patient (3.7%) 2 points (mean: 0.30 ± 0.53). Engorgement of the venous sinuses have a higher discriminative power than effacement of the suprasellar cistern (SSC). The prepontine cistern (PPC) was eliminated from the calculation of the SVM score due to its low specificity.

The mean SVM score was 1.41 (±0.60) for the SIH and 0.30 (±0.53) for the non-SIH patients (Mann-Whitney U‑test, *p* < 0.001). The area under the curve (AUC) of the receiver operating characteristic (ROC) curve was 0.91 (Fig. 3b). For comparison, we reviewed the structured reports of the 34 SIH patients which showed a good agreement between the SVM score and the results from the human reading as can be seen in the confusion matrix (Table 3).

**Table 3:** SVM score vs. results from human reading (HR) for the validation cohort

	SVM score = 0	SVM score = 1	SVM score = 2
HR = 0	*1* (2.9%)	*2* (5.9%)	*0* (0%)
HR = 1	*1* (2.9%)	*9* (26.4%)	*5* (14.7%)
HR = 2	*0 *(0%)	*5* (14.7%)	*11* (32.4%)

Confusion matrix for support vector machine (SVM) score compared to the results from the human reading (HR): 21 patients (61.8%) had the same score in human reading and SVM score, 13 patients (38.2%) had a score differing by one point, no patients had a score differing by 2 points. The mean score from human reading was 1.38 ± 0.64 and the mean score of the SVM algorithm was 1.41 ± 0.60 (*p* = n. s.)

## Discussion

In this study, we showed the feasibility of an automated approach of classifying head MRI scans to identify patients with spontaneous intracranial hypotension (SIH). A single MRI sequence, namely a contrast-enhanced T1-weighted MPRAGE sequence, which is part of our standard MRI protocol was analyzed [11]. As the segmentation of the considered volumes (superior sagittal sinus, both transverse sinuses, suprasellar cistern) is done by a computer there is no variability between raters allowing for an objective and unbiassed MRI assessment. This could be e.g. helpful in the follow-up of SIH patients and whether they show a benefit from treatment such as epidural blood patches.

Patients with proven CSF leaks in the validation cohort had as underlying causes ventral dural tears, meningeal diverticulae, and CSF venous fistulas in a proportion similar to previous reports [12, 13].

In contrast to the 9‑point score proposed by Dobrocky et al. which has been termed the Bern score later on [8, 14], we used a 2-point score. This simplification is due to the facts that effacements of the interpeduncular and prepontine cisterns are not accurately identified by the SVM algorithm and the trained CNN cannot accurately segment subdural effusions and pachymeningeal enhancement yet; however, there are other anatomical structures such as the pituitary gland which are more easily to segment and likely have good discriminating radiomic features (e.g. its superior contour) deserving further training and validation.

A limitation of our study is that the SVM algorithm was trained with structured reports of 19 independent neuroradiological physicians with an unknown intrarater and interrater variability; however, if only patients with proven CSF leaks and healthy controls would have been used for training, the area under the curve (AUC) of the receiver operating characteristic (ROC) analysis would likely be higher. So, training with a higher number of proven CSF leaks and analyzing additional anatomic structures responsive to CSF volume changes are future tasks.

A limitation of any automated approach is that it will fail to detect patients with (MRI signs of) intracranial hypertension who suddenly develop a CSF leak. Moreover, whether patients with very severe brain sagging are identified is not clear yet.

## Conclusion

We propose a simple, fully automatic SVM algorithm that analyzes contrast-enhanced MPRAGE sequences to identify SIH patients with a high diagnostic accuracy. It may help to consider the need of invasive diagnostics and transfer to a SIH center.

## Supplementary Information

Suppl Fig. 1: Dice coefficients of manual and convolutional neural network (CNN) segmentations for straight sinus (SR), superior sagittal sinus (SSS), left and right transverse sinuses (ST‑L and ST-R), interpeduncular cistern (IPC), prepontine cistern (PPC), and suprasellar cistern (SSC). The thresholds with the highest Dice coefficients are used for extraction of radiomic features.

Suppl Fig. 2: Volume (a) and Surface to volume ratios (b) normalized in z‑scales for superior sagittal sinus (SSS), left and right transverse sinuses (ST‑L and ST-R) and dominant transverse sinus (ST-D)

Suppl Fig. 3: Volume and least axis length normalized in z‑scales for suprasellar cistern (SSC)

## Abbreviations

CNN: Convolutional neural network
CSF: Cerebrospinal fluid
GT: Ground truth
IPC: Interpeduncular cistern
PPC: Prepontine cistern
SIH: Spontaneous intracranial hypotension
SSC: Suprasellar cistern
SVM: Support vector machine
