# Supplementary material for: Support Vector Machine-based Spontaneous Intracranial Hypotension Detection on Brain MRI
Source: Clin Neuroradiol. 2021 Oct 19;32(1):225–30. doi: 10.1007/s00062-021-01099-x (PMC8894221; doi:10.1007/s00062-021-01099-x)
Supplement: Supplementary file 1 — Suppl Fig. 1: Dice coefficients of manual and convolutional neural network (CNN) segmentations for straight sinus (SR), superior sagittal sinus (SSS), left and right transverse sinuses (ST‑L and ST-R), interpeduncular cistern (IPC), prepontine cistern (PPC), and suprasellar cistern (SSC). The thresholds with the highest Dice coefficients are used for extraction of radiomic features. [file 62_2021_1099_MOESM1_ESM.docx]

b

a

**Suppl Fig. 1** Dice coefficients of manual and CNN segmentations for straight sinus (SR), superior sagittal sinus (SSS), left and right transverse sinuses (ST-L and ST-R), interpeduncular cistern (IPC), prepontine cistern (PPC), and suprasellar cistern (SSC). The thresholds with the highest Dice coefficients are used for extraction of radiomic features
