# Supplementary material for: Support Vector Machine-based Spontaneous Intracranial Hypotension Detection on Brain MRI
Source: Clin Neuroradiol. 2021 Oct 19;32(1):225–30. doi: 10.1007/s00062-021-01099-x (PMC8894221; doi:10.1007/s00062-021-01099-x)
Supplement: Supplementary file 2 — Suppl Fig. 2: Volume (a) and Surface to volume ratios (b) normalized in z‑scales for superior sagittal sinus (SSS), left and right transverse sinuses (ST‑L and ST-R) and dominant transverse sinus (ST-D) [file 62_2021_1099_MOESM2_ESM.docx]

a

b

**Suppl Fig. 2** Volume (a) and Surface to volume-ratios (b) normalized in z-scales for superior sagittal sinus (SSS), left and right transverse sinuses (ST-L and ST-R) and
dominant transverse sinus (ST-D)
