# Supplementary material for: Support Vector Machine-based Spontaneous Intracranial Hypotension Detection on Brain MRI
Source: Clin Neuroradiol. 2021 Oct 19;32(1):225–30. doi: 10.1007/s00062-021-01099-x (PMC8894221; doi:10.1007/s00062-021-01099-x)
Supplement: Supplementary file 3 — Suppl Fig. 3: Volume and least axis length normalized in z‑scales for suprasellar cistern (SSC) [file 62_2021_1099_MOESM3_ESM.docx]

**Suppl Fig. 3** Volume and least axis length normalized in z-scales for suprasellar cistern (SSC)
